# Propofol prevents human umbilical vein endothelial cell injury from Ang II-induced apoptosis by activating the ACE2-(1-7)-Mas axis and eNOS phosphorylation

**DOI:** 10.1371/journal.pone.0199373

**Published:** 2018-07-11

**Authors:** Liangqing Zhang, Jingjing Wang, Jiuqing Liang, Du Feng, Fan Deng, Yue Yang, Yue Lu, Zhe Hu

**Affiliations:** 1 Department of Anesthesiology, Affiliated Hospital of Guangdong Medical University, Zhanjiang, China; 2 Guangdong Key Laboratory of Age-related Cardiac-cerebral Vascular Disease, Institute of Neurology, Affiliated Hospital of Guangdong Medical University, Guangdong Medical University, Zhanjiang, Guangdong, China; Max Delbruck Centrum fur Molekulare Medizin Berlin Buch, GERMANY

## Abstract

Angiotensin II (AngII), a vasoactive peptide that elevates arterial blood pressure and results in hypertension, has been reported to directly induce vascular endothelial cell apoptosis. Recent work has demonstrated that propofol pre-treatment attenuates angiotensin II-induced apoptosis in human coronary artery endothelial cells. However, the underlying mechanism remains largely unknown. Here, we investigated human umbilical vein endothelial cells (HUVECs) subjected to angiotensin II-induced apoptosis in the presence or absence of propofol treatment and found that angiotensin II-induced apoptosis was attenuated by propofol in a dose-dependent manner. Furthermore, ELISA assays demonstrated that the ratio of angiotensin (1–7) (Ang (1–7)) to Ang II was increased after propofol treatment. We examined the expression of ACE2, Ang (1–7) and Mas and found that the ACE2-Ang (1–7)-Mas axis was up-regulated by propofol, while ACE2 overexpression increased phosphorylated endothelial nitric oxide synthase (phosphorylated eNOS) expression and siACE2 resulted in the repression of endothelial nitric oxide synthase (eNOS) phosphorylation. In conclusion, our study revealed that propofol can inhibit endothelial cell apoptosis induced by Ang II by activating the ACE2-Ang (1–7)-Mas axis and further up-regulating the expression and phosphorylation of eNOS.

## Introduction

The vascular endothelium, a barrier between the vascular wall and the bloodstream, plays an important role in maintaining cardiovascular homeostasis [1]. Endothelial apoptosis is a pivotal risk factor for cardiovascular disease, and participates in the pathological development of vascular injury through endothelial dysfunction, directly resulting in cardiovascular dysfunction [2].

The renin-angiotensin-aldosterone system (RAAS) [3, 4] is thought to play a vital role in regulating vascular function and pathological responses to vascular injury. Ang II, a major active peptide of the RAAS, functions in modulating vascular tone and structure [5, 6]. However, AngII has been reported to directly induce vascular endothelial cell apoptosis [7], which aggravates vessel injury by binding the Ang II type 1 receptor (AGTR1) [8, 9]. Recent studies showed that Ang II-induced endothelial apoptosis is related to the stimulation of excessive reactive oxygen species (ROS) production and oxidative stress reactions to reduce the expression of endothelial nitric oxide synthase (eNOS) [10, 11]. eNOS is key to the generation of NO. Known as an ‘endothelium-derived relaxing factor’, NO produced by eNOS in the vascular endothelium not only maintains constant vascular tension but also has antioxidant, anti-inflammatory and anti-proliferative effects on vascular smooth muscle [12].

Recently, new members of the RAAS such as angiotensin converting enzyme 2 (ACE2) [7, 10, 13, 14], angiotensin (1–7) (Ang (1–7)) and Mas were identified. ACE2 is mainly expressed in endothelial cells and catalyses the conversion of AngII to Ang (1–7), which mediates effects that are opposite to those of AngII, with the consequent binding of Mas [2]. Ang (1–7) functions in vasodilation, as an anti-inflammatory and anti-proliferative agent, and in protecting against ischemia-reperfusion injury [2]. Additionally, Ang (1–7) activates protein-tyrosine phosphatases to suppress the MAPK activation and inhibits the generation of reactive oxygen species (ROS) [11, 15].

Propofol is widely used in clinical anaesthesia and in other fields as a commonly used intravenous anaesthetic. Propofol is structurally similar to the endogenous antioxidant α-Tocopherol (vitamin E) and possesses anti-inflammatory [11, 15–18] properties. Recent research showed that propofol suppresses oxidative stress-mediated endothelial cell dysfunction induced by hydrogen peroxide [19], tumour necrosis factor-α (TNF-α) [5]and ischemia-reperfusion injury [6]. In addition, propofol has been demonstrated to prevent nitrative stress and TNF-α-mediated endothelial cell activation and dysfunction induced by increased generation of endogenous NO [8]. However, it is still unclear whether propofol exerts a protective effect in Ang II-induced endothelial dysfunction. In this study, we prepared HUVEC model with Ang II treatment and investigated the potential mechanisms of propofol against Ang II-induced vascular endothelial cells injury and oxidative stress.

## Materials and methods

### Reagents and antibodies

Regents include propofol (Sigma-Aldrich, St Louis, MO, USA), dulbecco’s modified Eagle’s medium (DMEM), new-born calf serum (NCS), penicillin, streptomycin, trypsin-EDTA (GIBCO Laboratories, Grand Island, New York, USA), dimethylsulfoxide (DMSO), annexin V-FITC apoptosis detection kit, TUNEL detection kit, ELISA kit, and lipofectamine 3000 transfection reagent. Propofol was dissolved in DMSO and further diluted in phosphate buffered saline (PBS). The final DMSO concentration was 0.1%, which did not affect either cell function or the assay system (S1 Fig). The following primary antibodies were used in this study: beta-tubulin polyclonal antibody (Bioworld Technology, AP0064), Bcl-2 antibody (Abcam, ab32124), cleaved-caspase-9 antibody (CST, 7237), caspase-9 antibody (PTG, 66169-1-lg), eNOS antibody (Bioworld Technology, BS6837), phosphorylated eNOS antibody (Bioworld Technology, BS4848), anti-cytochrome C antibody (Abcam, ab13575), anti-angiotensin converting enzyme 2 (Abcam, ab15348), anti-MAS1 antibody (GeneTex, GTX45868), and AGTR1 antibody (PTG, 66415-1-lg). The above-mentioned primary antibodies are diluted to the appropriate concentration ratio (1:500~1:1000) with a proprietary anti-dilution solution (Bio Sharp, BL506A) according to the instructions.

### HUVEC culture and sample preparation

HUVECs were obtained from the type culture collection of the chinese academy of sciences, Shanghai, China. Briefly, HUVECs were cultured at 37°C, in a 95% O_2_ and 5% CO_2_ humidified atmosphere in DMEM supplemented with 20% foetal bovine serum, 100 μg/mL streptomycin, and100 IU/mL penicillin. For sample preparation, the culture media was replaced with glucose- and serum-free DMEM, with AngII added to the medium at different concentrations (10^−7^, 10^−6^, 10^−5^, 10^-4^M), and incubated in a normal incubator with 5% CO_2_ and 95% N_2_ for 24 h, after which the HUVECs were then returned to the maintenance medium (NCS-DMEM). At the same time, prepared propofol, together with The Mas receptor antagonist A779 and eNOS inhibitor NG-nitro-L-arginine methyl ester (L-NAME), was added to the medium at different concentrations (50μmol/L-150μmol/L) for 4 h. We used H_2_O_2_ as another inducer for apoptosis [17, 19], the proper concentration of which was set to 400μM (S2 Fig).

### Flow cytometry

Cells were treated with angiotensin II for 24 hr, followed by propofol for 4 hr, and on the day of the experiment, they were trypsinized, washed once with ice-cold PBS and stained with annexin V and propidium iodide using a specific kit (BD Biosciences, Franklin Lakes, NJ, USA). Flow cytometry analysis of 20 000 cells per sample was performed on a COULTER EPICS XL-MCLTM flow cytometer (Beckman Coulter, Fullerton, CA, USA) and quantified using EXPO32 ADC analysis software (Beckman Coulter).

### TUNEL assay

The presence of death-associated DNA fragmentation was assessed in situ by terminal deoxynucleotidyl transferase (TdT)-mediated dUTP nick end labelling (TUNEL). On day two after plating, 50% confluent cells grown on coverslips were incubated with Ang II as indicated. After 24 h, cells were treated with different concentrations of propofol for another 4 h, after which cells were fixed with Triformol for 10 min, and equilibration buffer was directly applied on the cells for 10 min at room temperature. TdT enzyme was mixed in reaction buffer and applied to the cells for 1 h at 37°C. Next, 2×SSC (saline-sodium citrate buffer) was added to the coverslips and incubated at room temperature for 15 min. Following PBS washes, diaminobenzidine (DAB) peroxidase substrate was applied, and cells were counterstained with 6-diamidino-2-phenylindole (DAPI). Cells were photographed on a Leica TCS SP5Ⅱ microscope.

### Measurement of ROS and NO

The level of ROS was determined as oxidative stress. HUVECs were stimulated by Ang II and propofol as described, incubated with DCFH-DA for 40 min at 37°C for measurement. ROS production in HUVECs was detected using a reactive oxygen species assay kit to the manufacturer's instructions (Nanjing Jiancheng Bioengineering Institute, China). The fluorescence intensity was detected using a fluoresce microplate reader with the wavelength at 525nm.

HUVECs were treated with Ang II in the absence or presence of propofol. NO production in medium was detected using a nitric oxide assay kit (Nitrate reductase method) according to the manufacturer's instructions (Nanjing Jiancheng Bioengineering Institute, China). The fluorescence intensity was detected with the wavelength at 520-560nm.

### Western blotting

The expression of apoptosis- and ACE2-Ang (1–7)-Mas axis-related proteins were determined by Western blotting. Whole-cell lysates were prepared in RIPA buffer (50 mM Tris-HCl, pH 8, 150 mM NaCl, 1% NP-40, 0.1% SDS, and 1% Triton X-100 plus proteinase inhibitors; Sigma). Protein concentrations were determined using the bradford assay, and samples containing 30 μg were separated via 12% SDS-PAGE. Proteins were transferred to nitrocellulose membranes, which were then blocked in 5% non-fat milk in 20mM Tris-HCl, 150 mM NaCl, and 0.05% Tween 20 for 1 h at room temperature, followed by overnight incubation with different primary monoclonal antibodies at 4°C; β-Tubulin antibody was used as a loading control. The membranes were then incubated with secondary antibody conjugated to horseradish peroxide for 1 h at room temperature and then exposed to enhanced chemiluminescence reagents. Densitometric analysis was performed to quantify signal intensity.

### Measurement of the concentration of Ang II and Ang (1–7)

ELISA assay kit for Ang (1–7) /Ang II was purchased from CUSABIO Company (cat. no.CSB-E04493h). The detection range of human angiotensin 1–7 (Ang1-7) ELISA kit is 7.8pg/ml-500pg/ml, and the minimum detectable dose of human Ang1-7 is typically less than 1.95pg/ml. The detection range of human Ang II ELISA kit is 31.25pg/ml-2000pg/ml, and the minimum detectable dose of human Ang II is typically less than 7.8pg/ml. Cell culture supernates were centrifuged for 15 minutes at 1000 x g at 2–8°C to remove particulates and assay immediately. Standards and samples are pipetted into the antibody pre-coated microplate and incubated for 2 hours at 37°C, after washing, avidin conjugated horseradish peroxidase (HRP) and biotin-antibody are added into the wells and incubated for 1 hour at 37°C. The TMB substrate is added and colour develops in proportion to the amount of Ang (1–7) /Ang II, the absorbance of each sample was measured by enzyme-linked immunosorbent analyser with the readings at 450 nm. The content of Ang II or Ang (1–7) was calculated in accordance with the standard curve.

### Transfections and siRNA interference for ACE2

Human ACE2 siRNA (5’- GAGGAGACUAUGAAGUAAATT-3’), (5’- UUUACUUCAUAGUCUCCUCTT -3’) and negative control siRNA (GenePharma, China) were transfected using lipofectamine 3000 (Invitrogen, Carlsbad, USA) following the manufacturer’s protocol. siRNAs and plasmids were evaluated with western blot (S3 Fig). For ACE2 overexpression, HUVECs were transfected with plasmid DNA carrying the transcriptional target sequence of ACE2 promoter sequences together with a GFP reporter vector used to normalize experimental variations, including transfection efficiency. Cells were harvested 12 h later and lysed for western blotting. All transfections were performed in triplicate, and three independent experiments were similarly performed.

### Statistical analysis

Statistical comparisons were made using the Kruskal Wallis test in SPSS V11.0 software. P<0.05 was considered significant.

## Results

### Protective effects of propofol in AngII-induced apoptosis

**Fig 1:**
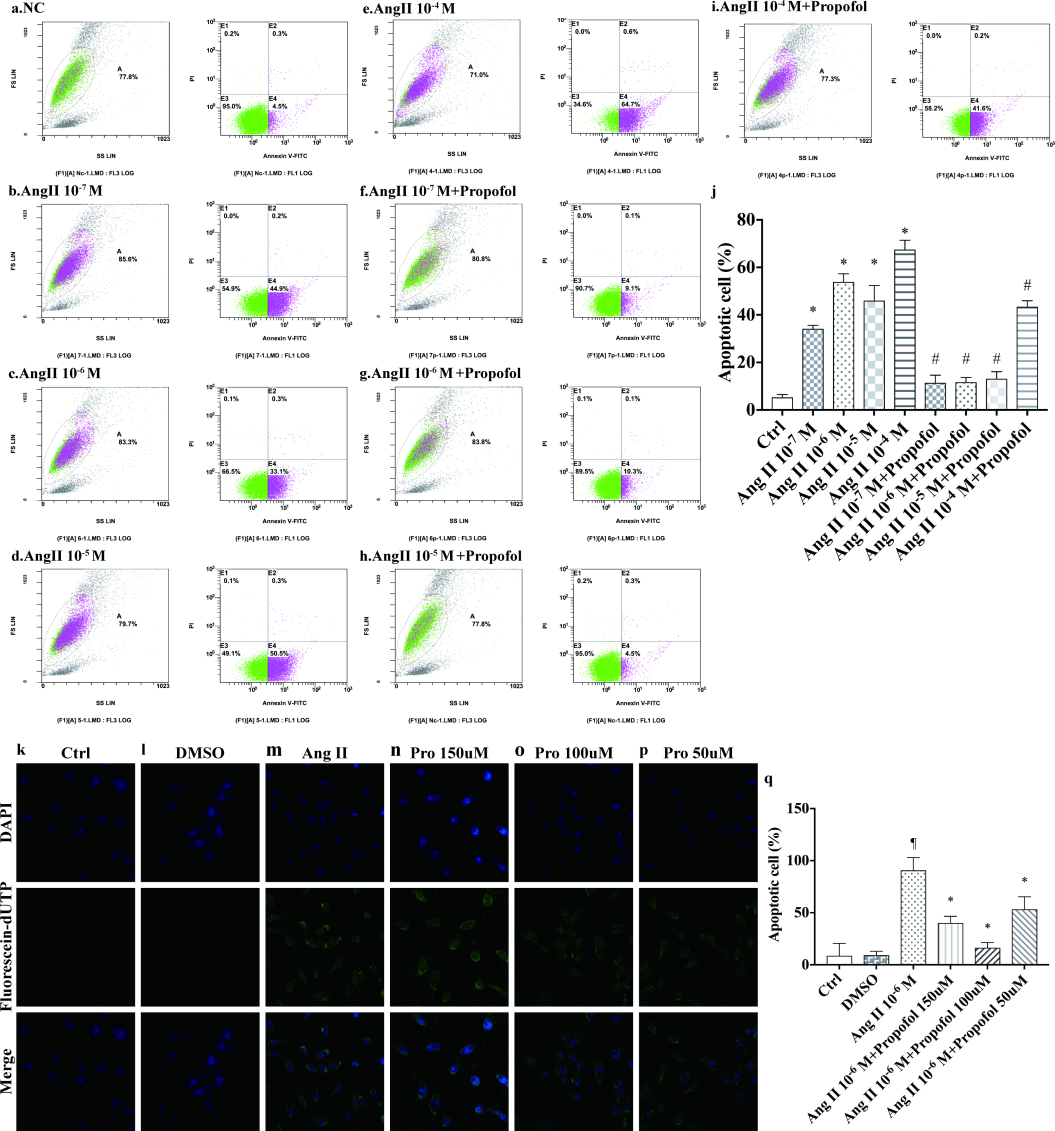
Propofol inhibits AngII-induced apoptosis in HUVECs. (a-j) Detection of apoptosis with Annexin V-FITC and propidium iodide staining. Cells were preconditioned with increasing concentrations of AngII (10^−7^–10^-4^M) for 24 h and then treated with or without propofol for 4 h. Each group of Annexin V- and propidium iodide-stained cells was measured by flow cytometry. The histogram represents the percentage of apoptotic cells, and the data represent the mean±s.d. of three independent experiments. (n = 3) *P<0.01 versus the control group and # P<0.05 versus corresponding concentration AngII injury groups (k-q). AngII-pre-treated HUVECs were treated with different concentrations (0–150μmol/L) of propofol for 4 h. Cell apoptosis was evaluated via TUNEL assay. Data are from n = 3 independent experiments. ¶versus the DMSO group (P<0.01) and *versus the AngII injury group (P<0.01).

To confirm the inhibitory effect of propofol on AngII-induced cell apoptosis, HUVECs were stained with annexin V-FITC and propidium iodide, then analysed by flow cytometry. The percentage of apoptosis in the control groups was 5.31±1.15%. The fraction of apoptotic cells increased to34.15±3.4%, 52.9±13.9%,46.05±6.3%, and 67.5±3.9% when cells were treated with 10^−7^, 10^−6^, 10^−5^, and 10^-4^M AngII, respectively, compared with the control group (P<0.05versus control group). Propofol treatment resulted in reduced AngII-induced cell apoptosis (Fig 1A–1J). The percentage of apoptotic cells decreased to11.4±3.25%, 11.7±2.0%, 13.18±3.0%, and 43.4±2.54% at 10^−7^, 10^−6^, 10^−5^, and 10^-4^M AngII, respectively (P<0.01 versus AngII group) (Fig 1A–1J). According to these results, we choose 10^−6^ M AngII for subsequent studies. TUNEL assays were performed to find the most effective concentration of propofol against AngII-induced cell apoptosis. (Fig 1K–1Q) The percentage of apoptotic cells was 9±0.12% and 9±0.39% in the control and DMSO groups (to exclude the possibility of solvent effects, 0.1% DMSO was added to the medium). Addition of AngII to cells resulted in accelerated cell apoptosis (91±0.12%). Cell apoptosis decreased in all of the propofol-treated groups compared with the AngII group (53±0.12% and16±0.05% at 50 and 100μmol/L propofol, respectively) (P<0.01), except for the 150μmol/L group (40±0.06%), indicating that administration of excessive propofol has no extra protective effects.

### Propofol inhibits AngII-induced apoptosis by up-regulating the ACE2-Ang (1–7)-Mas axis

**Fig 2:**
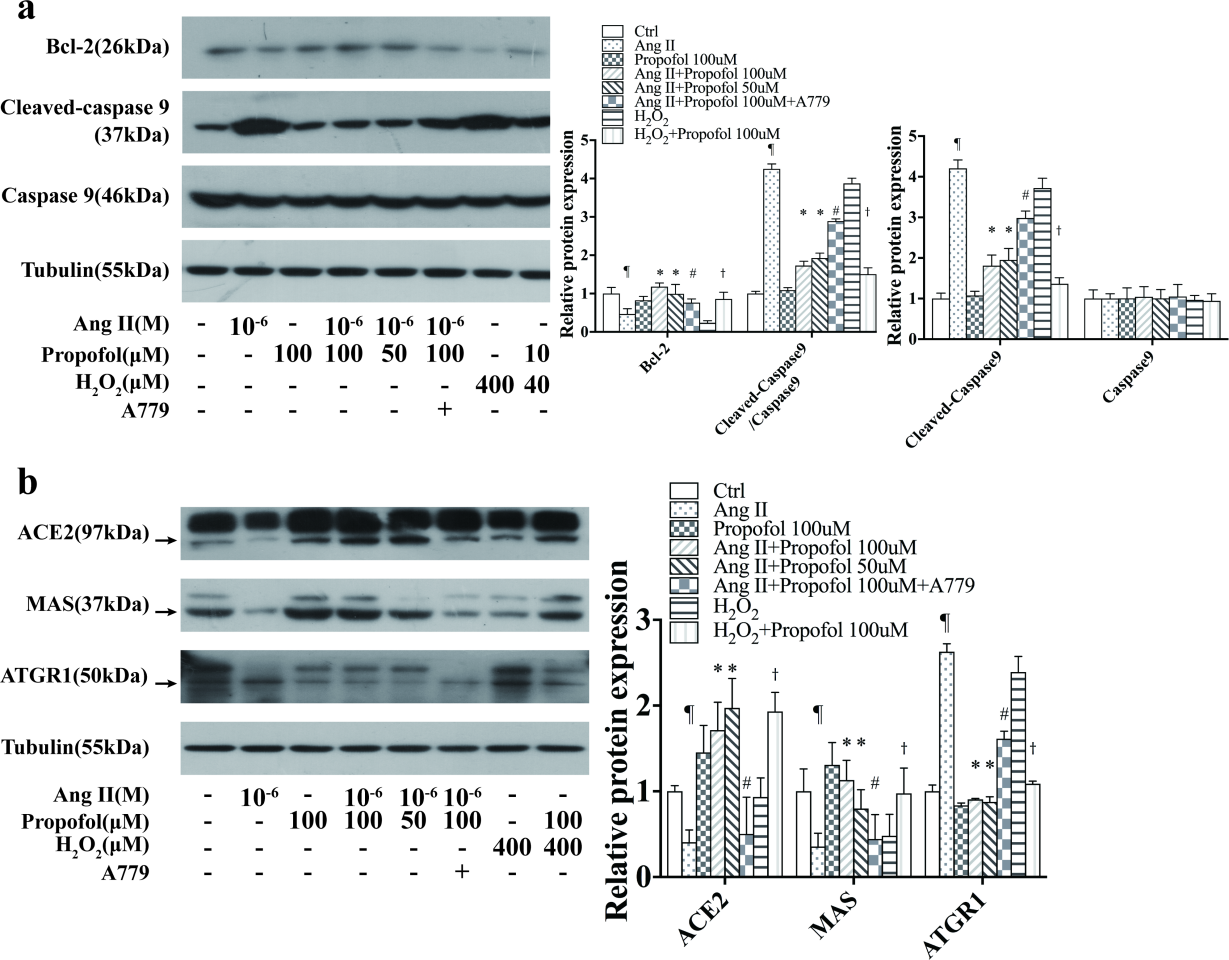
Propofol inhibits AngII-induced apoptosis by up-regulating the ACE2-Ang (1–7)-Mas axis. (a) Western blotting was performed to evaluate the expression of the apoptosis-related proteins caspase-9 and Bcl-2. The expression of cleaved caspase-9 decreased and that of Bcl-2 was significantly increased after propofol treatment for 4 h compared with the control group. After A779 administration, the expression of cleaved caspase-9 was down-regulated and Bcl-2 levels increased. (b) ACE2 and Mas protein levels were up-regulated; however, ATGR1 protein level was decreased in the propofol group compared with the control group. A779 decreased the protein levels of ACE2 and Mas while up-regulated ATGR1 after A779 treatment. The data are representative of six independent experiments, *P<0.01 versus the AngII -treated group, # P<0.01 versus the AngII+propofol 100μM group, ¶P<0.01 versus the control group, and †P<0.01 versus the H_2_O_2_ -treated group.

AngII can directly lead to vascular endothelial cell apoptosis; however, Ang (1–7) play an opposing role to that of AngII [11]. To determine whether Ang (1–7) is involved in the suppression of AngII induced apoptosis by propofol, we used the Mas receptor antagonist A779 to inhibit Ang (1–7) binding to Mas. We then examined the apoptosis-related proteins by western blotting and found increased levels of cleaved caspase-9, the active form of caspase-9, whereas the anti-apoptotic protein Bcl-2 was down-regulated upon AngII treatment (Fig 2A). However, caspase-9 activation was blocked, and Bcl-2 expression increased in response to increasing concentrations of propofol. Notably, in the presence of A779, the reduction on Bcl-2 and cleaved caspase 3 by propofol were reversed. These data demonstrate that propofol protected AngII-induced apoptosis involves Mas receptor.

The ACE2-Ang (1–7)-Mas axis was recently described as a novel component of the RAAS, in which ACE2 hydrolyses AngII to form Ang (1–7) [2]. To clarify whether propofolup-regulatesACE2 and Ang (1–7) to prevent AngII-induced apoptosis, we monitored the expression of ACE2 and Mas (Fig 2B) after propofol treatment. As expected, AngII treatment resulted in the down-regulation of ACE2, slight decrease in Mas expression and up-regulation of AGTR1, indicating the suppression of Ang (1–7) expression and the activation of AngII- AGTR1 pathway. After propofol treatment, ACE2 levels were up-regulated and the expression of Mas receptor and AGTR1 recovered to a similar level as the control, this effect was blocked by the Mas receptor antagonist A779.

### Propofol increases Ang (1–7) levels

**Fig 3:**
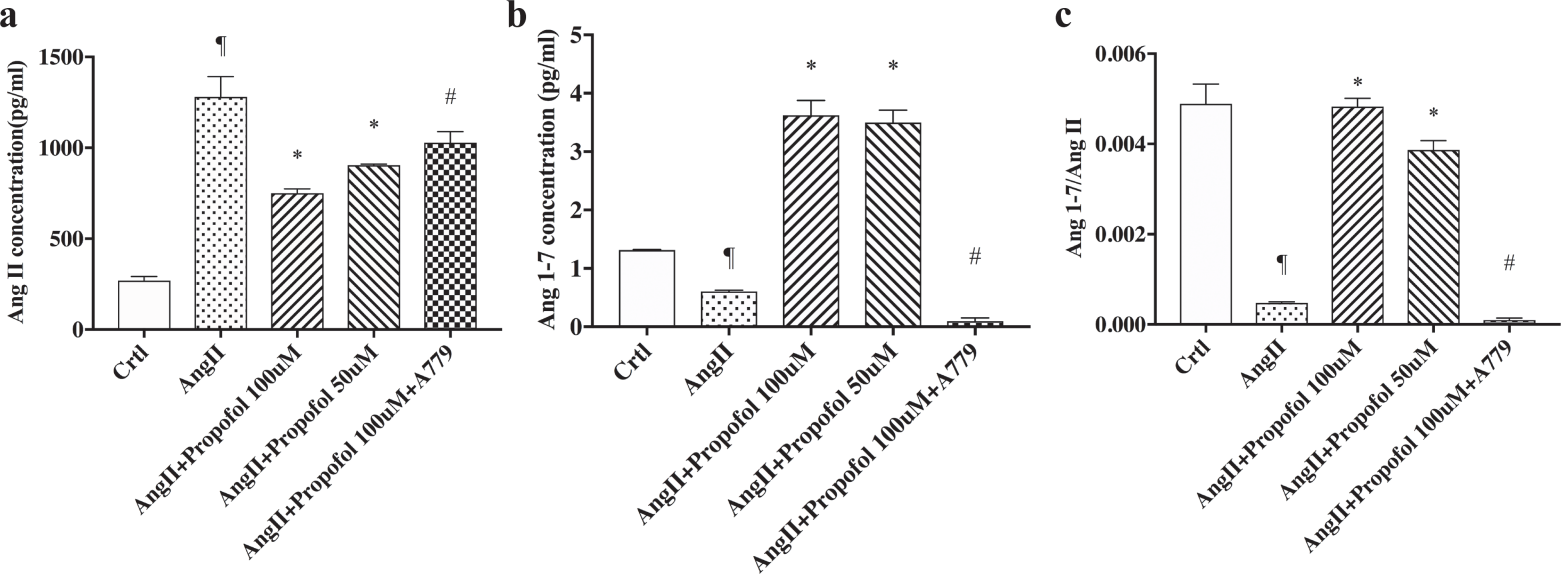
Propofol up-regulates the expression of Ang (1–7), as quantified by ELISA. HUVECs cultured in 96-well plates were treated with AngII for 24 h, followed by treatment with propofol with or without A779 for 4 h. Data are shown as the mean±s.d. from n = 3 independent experiments. (a) The level of Ang (1–7) was quantified by ELISA; *P<0.01 versus the AngII group, and #P<0.05 versus the control group. (b) The level of AngII was quantified by ELISA using a commercial kit. # P<0.05 versus the AngII group. (c) The ratio of Ang (1–7) to AngII expression was calculated and shown as (c). *P<0.01 versus the AngII -treated group, # P<0.05 versus the AngII+propofol 100μM group, and ¶P<0.01 versus the control group.

The above data suggest that propofol treatment results in ACE2 up-regulation to hydrolyse AngII to form Ang (1–7). To confirm that propofol directly contributes to the generation of Ang (1–7), we performed ELISA to evaluate the AngII/Ang (1–7) ratio after propofol treatment. The AngII group was used to exclude solvent on against apoptosis. As shown in Fig 3, Ang (1–7) was up-regulated after propofol treatment; however, no significant difference was found in AngII expression compared with the control. The ratio of Ang (1–7) to AngII expression was calculated, and the data distinctly reflects a dose-dependent increase in Ang (1–7)/AngII with propofol treatment.

### Propofol inhibited Ang II-mediated oxidative damage

Oxidative stress is accepted as a critical pathogenic factor in endothelial cell dysfunction. In order to elucidate whether propofol contribute to inhibiting Ang II -mediated oxidative damage, we investigated some oxidative stress related indicators such as ROS and NO.

**Fig 4:**
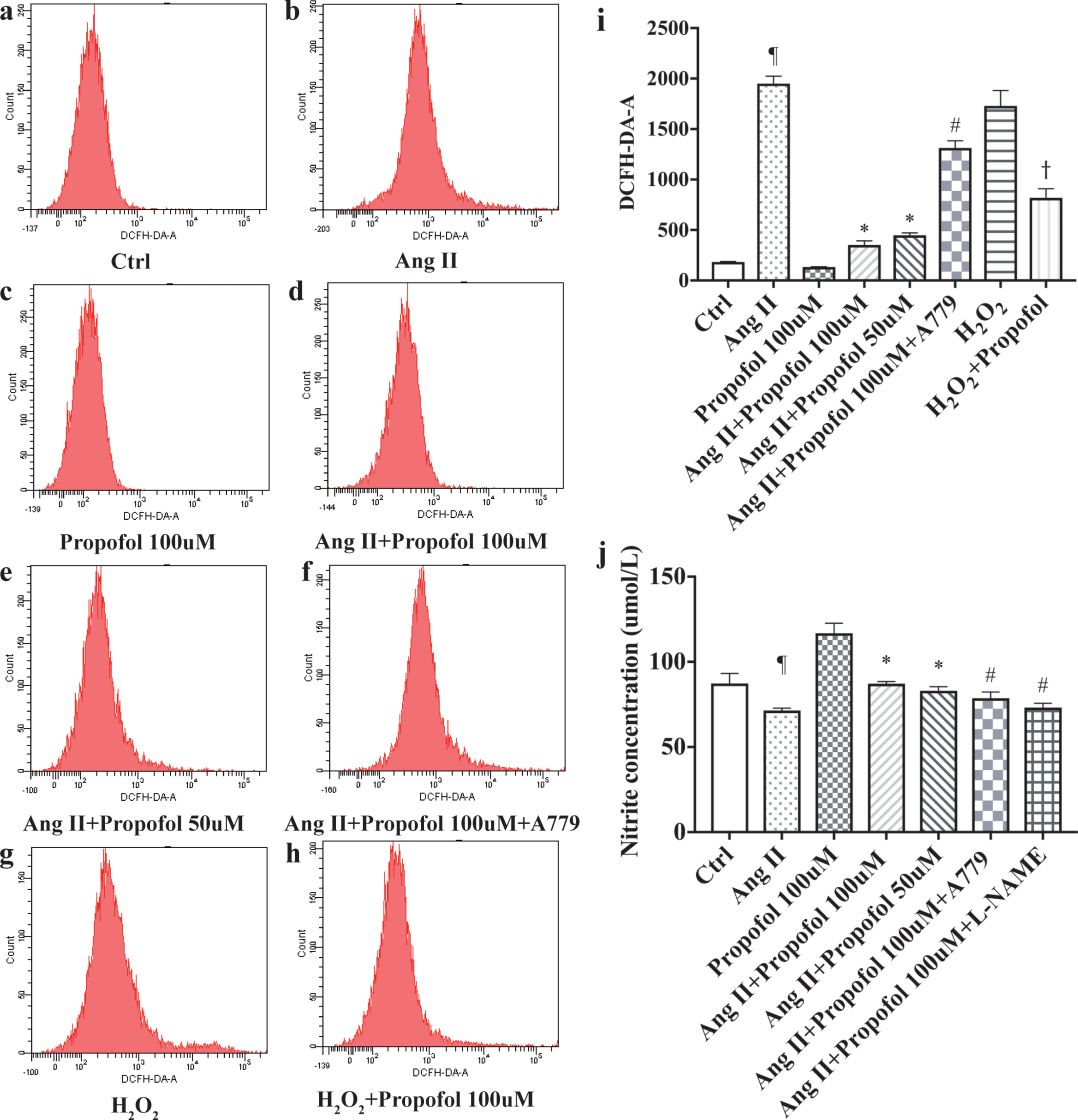
Propofol inhibited Ang II-mediated oxidative damage. (a–h) ROS assay showed that treatment with propofol reduced Ang II-stimulated ROS formation. (i–j) NO assay showed that Addition of propofol exhibited higher NO production. The results are shown as the mean ± SD (n = 3). ¶ p<0.05 compared to the Ctrl group; * p<0.05 compared to the Ang II group; # p<0.05 compared to the Propofol 100uM group; and † P<0.05 compared to H_2_O_2_ group.

Treatment with Ang II led to a sharp augment in ROS generation (Fig 4A–4I), which was reduced by propofol, whereas the effect was counteracted when A779 is present. By contrast, Ang II treatment resulted in a decrease in NO production compared with control. The addition of propofol reversed the inhibitory effects of Ang II on NO production (Fig 4J). A779 and eNOS inhibitor L-NAME slightly decreased the level of NO. These data suggested that propofol inhibited Ang II -mediated oxidative damage in HUVECs, which might be benefit in attenuating endothelial dysfunction.

### Effect of propofol on AngII-induced apoptosis through eNOS phosphorylation

**Fig 5:**
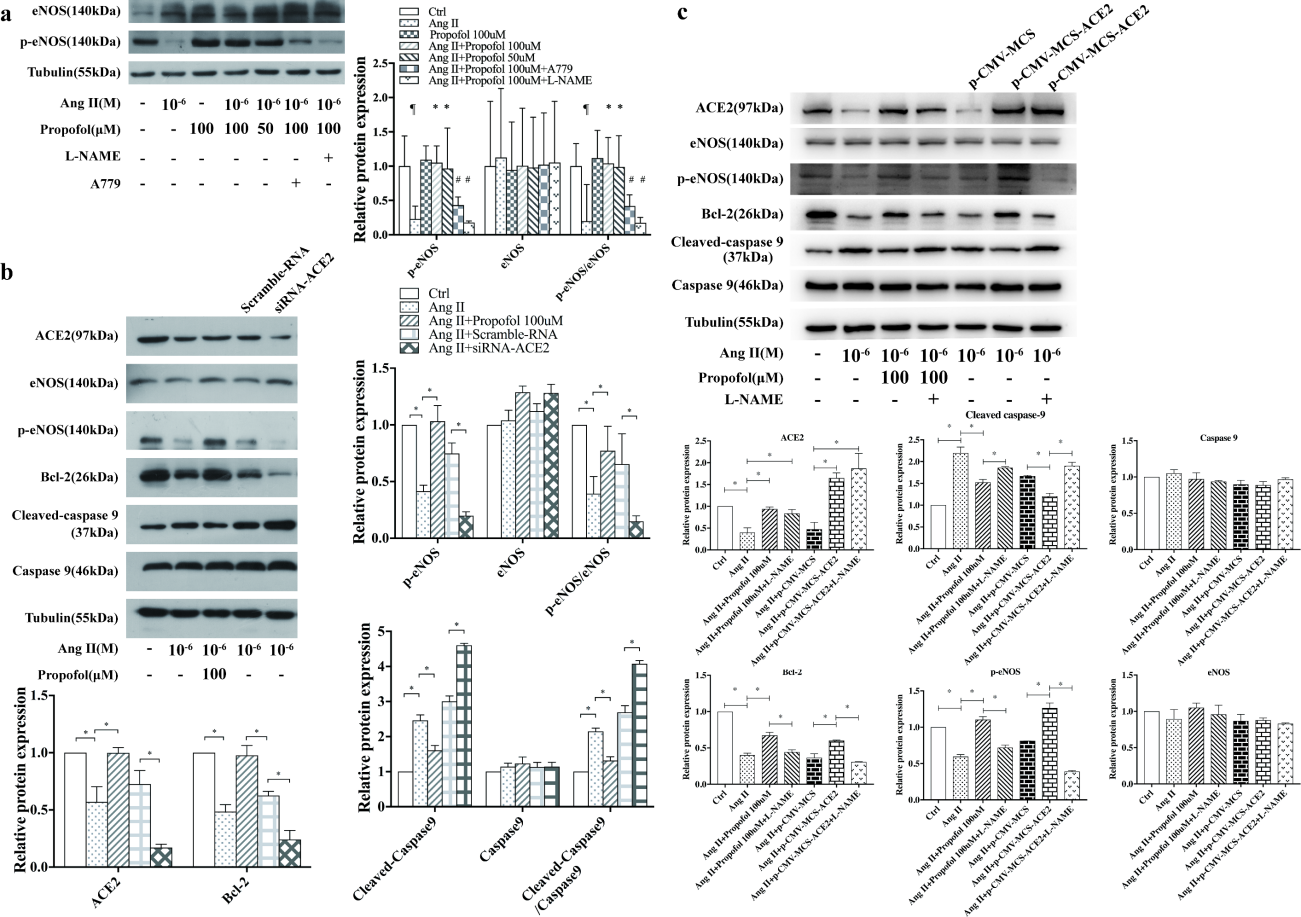
Propofol prevents AngII-induced apoptosis by regulating ACE2 and phosphorylated eNOS. (a) A representative Western blot of eNOS activation in AngII-pre-treated HUVECs among normal (Control), propofol-treated and with or without A779 and L-NAME addition after propofol treatment for 4 h. Following the process described in the Materials and Methods, equal amounts of total protein from each cell sample were subjected to Western blotting using specific antibodies against eNOS and phosphorylated eNOS. β-Tubulin was used to confirm equal protein loading. The results are shown as the mean ± SD (n = 3). ¶ p<0.05 compared to the Ctrl group; * p<0.05 compared to the Ang II group; # p<0.05 compared to the Propofol 100uM group. (b) Expression of phosphorylated eNOS in HUVECs in AngII-treated medium, with or without propofol-treatment. HUVECs transfected with siRNA-ACE2 and scramble-RNA were harvested 48h after transfection. Data are representative of n = 3 independent WB experiments; *P<0.01. (c) Expression of phosphorylated eNOS in HUVECs in AngII-treated medium, propofol-treated and with or without L-NAME addition after propofol treatment for 4 h. HUVECs transfected with an ACE2-overexpression plasmid were harvested 48h after transfection. Data are representative of n = 3 independent WB experiments; *P<0.01.

Studies have shown that AngII-induced endothelial apoptosis is mediated by the stimulation of ROS generation to reduce eNOS expression [9, 10]. We examined eNOS and phosphorylated eNOS by western blotting and found that the expression of phosphorylated eNOS was up-regulated following propofol treatment, whereas this effect was counteracted by L-NAME or A779 (Fig 5A). This result, therefore, indicated that eNOS phosphorylation may be an important step in the prevention of Ang II-induced apoptosis by propofol and that the ACE2-Ang (1–7)-Mas axis may also play a role in this process.

To clarify the effect of the ACE2-Ang (1–7)-Mas axis on eNOS phosphorylation, we knocked down and overexpressed ACE2 in HUVECs. The construction of the cellular transfection model was confirmed by the expression of GFP in ACE2-GFP via western blotting, and ACE2 expression was decreased in the siRNA-ACE2 group (scrambled- siRNA was used as a negative control) (Fig 5B) and increased in ACE2-GFP cells (Fig 5C). We then examined the expression of caspase-9 and found that cleaved caspase-9 levels increased in the siRNA-ACE2 group compared with the scrambled-siRNA group but decreased in the ACE2-GFP group compared with the GFP group, whereas the effect was counteracted when L-NAME is present. (P<0.05), indicating that ACE2 expression plays an important role in AngII-induced endothelial apoptosis. However, phosphorylated eNOS levels decrease in the siRNA-ACE2 group compared with the scrambled-siRNA group and increased in the ACE2-GFP group compared with the GFP group (P<0.05). The addition of L-NAME inhibited the Promoting effects of propofol on eNOS phosphorylation.

## Discussion

Endothelial cell dysfunction could be caused by numerous risk factors and is intimately linked to progression of acute respiratory distress syndrome [20], systemic inflammatory response syndrome, and multi-organ dysfunction syndrome. Hydrogen peroxide (H_2_O_2_) has been shown to cause severe cell dysfunction [21] in human umbilical vein endothelial cells (HUVECs). Alvarez E and colleagues reported that Ang II induces endothelial cell apoptosis by triggering NADPH oxidase to produce a large amount of ROS [22].

Propofol (2, 6-diisopropylphenol) is an intravenous sedative-hypnotic agent that provides an enhanced antioxidant effect and protection against oxidative damage both in vitro and in vivo [19, 23–25]. Chen et al. reported that propofol pre-treatment can attenuate angiotensin II-induced apoptosis in human coronary artery endothelial cells. Therefore, we hypothesized that propofol improves endothelial function and inhibit HUVEC apoptosis subjected to Ang II treatment. Our data demonstrate that propofol significantly inhibited HUVEC apoptosis, which is enhanced by Ang II. These results suggested the mechanism of the protective effect of propofol may be involved in suppressed apoptosis. We further examined the expressions of apoptosis related proteins bcl-2, caspase 9 and cleaved caspase 9 in order to explore the signalling pathway responsible for the protective effect of propofol and found propofol markedly up-regulated the bcl-2 expression and decrease the level of cleaved caspase-9. Thus, propofol may exert its suppressive effects on HUVEC apoptosis via regulation of bcl-2 and caspase-9.

Ang II, the biologically active key effector in the RAAS, is a generally accepted biomarker for predicting the progression of cardiovascular diseases [12]. The newly defined ACE2- Ang (1–7)-Mas axis was shown to contribute to causing vasodilation, anti-hypertrophy and improving vascular function, exerting the opposite actions of Ang II [26]. We hypothesized that endothelial dysfunction could be attenuated by propofol by modulating the balance of Ang II and Ang (1–7). ELISA assay demonstrated that propofol led to prominent conversion of Ang II to Ang-(1–7), with a reduction in the level of Ang II and an increase in Ang-(1–7), which indicated the recovery of endothelial function. ACE2, highly expressed in the vascular endothelium, efficiently metabolizes Ang II into a putatively protective peptide Ang-(1–7) [27, 28]. Hence, ACE2 has been accepted as a promising therapeutic target for Hypertension. Propofol has been shown to increase ACE2 in earlier study via a PI3K-dependent mechanism [29], so we postulated propofol can increase ACE2 to protect endothelial from dysfunction through catalysing the conversion of Ang II to Ang-(1–7). In the study, propofol improved ACE2 expression and ACE2 activity which were inhibited by Ang II. Besides, Mas expression was also increased, and AGTR1 was suppressed. Ang II can be activated when interacted with AGTR1 to play a central role in the up-regulation of blood pressure [30, 31]. However, Excessive activation of the ACE-AngII-ATIR pathway can directly cause endothelial injury [22]. Our data suggested propofol protect endothelial from apoptosis by increasing ACE2 to catalize Ang II to Ang-(1–7), resulting in suppression of the activation of the ACE-AngII-ATIR.

Evidence has shown that oxidative stress is considered to be a critical pathogenic factor responsible for endothelial cell dysfunction and the development of cardiovascular diseases [32, 33]. To inhibit or interfere with ROS generation and reducing ROS accumulation might be the potential ways to protect endothelial cells from injury. Propofol was reported to demonstrate antioxidant effects and shown to exhibit potent H_2_O_2_ [34] and hydroxyl radical [35] scavenging activity. Since Ang II is a potent activator of oxidative stress, we speculated that propofol treatment may lead to a reduction in oxidative stress to improve endothelial function. Our data demonstrated that propofol effectively attenuates oxidative stress by inducing lots of variation in some oxidative stress indicators in the Ang II-damaged HUVECs. Propofol decreased ROS generation and further stimulated NO production.

Endothelial cells play a role in the regulation of the vascular tone, by ways of generation of vasodilator such as nitric oxide (NO). Propofol was reported to regulate nitric oxide production derived from endothelium [36, 37], but the exact mechanism has not been fully elucidated. Rabelo LA and Alenina N found that Ang II reduces NO activity in endothelial cells by enhancing NADPH oxidase phosphorylation [26]. However, angiotensin-(1–7), by interacting with its receptor Mas, induces the release of NO from endothelial cells and thereby counteracts the effects of angiotensin II. Thus, we hypothesized that propofol up-regulates ACE2/Ang-(1–7)/Mas axis to protect against endothelial injury by modulating the release of NO. In ECs, NO is largely synthesized enzymatically by endothelial nitric oxide synthase (eNOS) [38]. Propofol was reported to increase phosphorylation of eNOS at Ser^1177^ for the NO production, which was involved in PKC activation and intracellular translocation [39, 40]. Thus, we explored the expression of eNOS and P-eNOS, indeed, propofol efficiently increased the expression of P-eNOS and the effect severely blocked by addition of Mas receptor antagonist A779 and L-NAME, suggesting that the NO generation was induced by propofol through eNOS phosphorylation and activation of MAS. To further explore the signalling pathway responsible for MAS activation, plasmids were transfected to knockout or overexpress ACE2. The result revealed that the eNOS phosphorylation was increased in an ACE2 dependent manner, and the up-regulation of ACE2 is responsible for the protective effect of propofol against epithelial apoptosis.

In conclusion, our study revealed that propofol significantly attenuates Ang II-induced endothelial dysfunction. The mechanism involved the up-regulation of ACE2-Ang (1–7)-Mas axis, subsequent phosphorylation of eNOS to generate NO, and regulation of apoptosis-related protein such as bcl-2, caspase9. The present study provides new insights into propofol’s protective effect against endothelial dysfunction as well as its therapeutic potential for hypertension.

## Supporting information

S1 Fig The effects of DMSO on HUVECs apoptosis. Cells were treated with or without 0.1% DMSO for 4h, and then cleaved-caspase 9, caspase 9, Bcl-2 were detected by Western blot analysis. The results are shown as the mean ± SD (n = 3).
(TIF)

S2 Fig Propofol promoted HUVECs proliferation. The cell viability was measured by CCK8 assay. HUVECs were incubated with different concentration of H2O2 for 4 h. The results are shown as means ± SD (n = 3). * P < 0.01, compared with control.
(TIFF)

S3 Fig siRNAs and plasmids were evaluated with western blot. Western blot showed ACE2 protein expression which was knockout or overexpressed. The results are shown as means ± SD (n = 3). * P < 0.01, compared with control.
(TIF)

S4 Fig Propofol increased ACE2 expression in a time-dependent manner. Western blot showed ACE2 protein expression which was treated with propofol in AngII injured HUVECs. The results are shown as means ± SD (n = 3). # P < 0.01, compared with control; * p<0.05 compared to the Ang II group.
(TIF)

## References

[1] Zicha J, Kunes J. Ontogenetic aspects of hypertension development: analysis in the rat. Physiol Rev. 1999;79(4):1227–82. Epub 1999/10/03. doi: 10.1152/physrev.1999.79.4.1227 . 10508234 10.1152/physrev.1999.79.4.1227

[2] Davignon J, Ganz P. Role of endothelial dysfunction in atherosclerosis. Circulation. 2004;109(23 Suppl 1):Iii27–32. Epub 2004/06/17. doi: 10.1161/01.CIR.0000131515.03336.f8 . 15198963 10.1161/01.CIR.0000131515.03336.f8

[3] Bader M, Ganten D. Update on tissue renin-angiotensin systems. J Mol Med (Berl). 2008;86(6):615–21. Epub 2008/04/17. doi: 10.1007/s00109-008-0336-0 . 18414822 10.1007/s00109-008-0336-0

[4] Bader M. Tissue renin-angiotensin-aldosterone systems: Targets for pharmacological therapy. Annu Rev Pharmacol Toxicol. 2010;50:439–65. Epub 2010/01/09. doi: 10.1146/annurev.pharmtox.010909.105610 . 20055710 10.1146/annurev.pharmtox.010909.105610

[5] Wang B, Shravah J, Luo H, Raedschelders K, Chen DD, Ansley DM. Propofol protects against hydrogen peroxide-induced injury in cardiac H9c2 cells via Akt activation and Bcl-2 up-regulation. Biochem Biophys Res Commun. 2009;389(1):105–11. Epub 2009/08/26. doi: 10.1016/j.bbrc.2009.08.097 ; PubMed Central PMCID: PMCPMC3631547. 19703415 10.1016/j.bbrc.2009.08.097 PMC3631547

[6] Le T, Hwang WL, Muralidhar V, White JA, Moore MS. First aid for the basic sciences 2017.

[7] Li D, Tomson K, Yang B, Mehta P, Croker BP, Mehta JL. Modulation of constitutive nitric oxide synthase, bcl-2 and Fas expression in cultured human coronary endothelial cells exposed to anoxia-reoxygenation and angiotensin II: role of AT1 receptor activation. Cardiovasc Res. 1999;41(1):109–15. Epub 1999/05/18. . 10325958 10.1016/s0008-6363(98)00196-5

[8] Mehta PK, Griendling KK. Angiotensin II cell signaling: physiological and pathological effects in the cardiovascular system. Am J Physiol Cell Physiol. 2007;292(1):C82–97. Epub 2006/07/28. doi: 10.1152/ajpcell.00287.2006 . 16870827 10.1152/ajpcell.00287.2006

[9] Montezano AC, Nguyen Dinh Cat A, Rios FJ, Touyz RM. Angiotensin II and vascular injury. Curr Hypertens Rep. 2014;16(6):431 Epub 2014/04/25. doi: 10.1007/s11906-014-0431-2 . 24760441 10.1007/s11906-014-0431-2

[10] Waghe P, Sarath TS, Gupta P, Kandasamy K, Choudhury S, Kutty HS, et al Arsenic causes aortic dysfunction and systemic hypertension in rats: Augmentation of angiotensin II signaling. Chem Biol Interact. 2015;237:104–14. Epub 2015/06/17. doi: 10.1016/j.cbi.2015.06.014 . 26079204 10.1016/j.cbi.2015.06.014

[11] Wang L, Wu B, Sun Y, Xu T, Zhang X, Zhou M, et al Translocation of protein kinase C isoforms is involved in propofol-induced endothelial nitric oxide synthase activation. Br J Anaesth. 2010;104(5):606–12. Epub 2010/03/30. doi: 10.1093/bja/aeq064 . 20348139 10.1093/bja/aeq064

[12] Liu X, Chen H, Zhan B, Xing B, Zhou J, Zhu H, et al Attenuation of reperfusion injury by renal ischemic postconditioning: the role of NO. Biochem Biophys Res Commun. 2007;359(3):628–34. Epub 2007/06/06. doi: 10.1016/j.bbrc.2007.05.129 . 17548062 10.1016/j.bbrc.2007.05.129

[13] Yee AH, Burns JD, Wijdicks EF. Cerebral salt wasting: pathophysiology, diagnosis, and treatment. Neurosurg Clin N Am. 2010;21(2):339–52. Epub 2010/04/13. doi: 10.1016/j.nec.2009.10.011 . 20380974 10.1016/j.nec.2009.10.011

[14] Robbins SL, Kumar V, Cotran RS. Robbins and Cotran pathologic basis of disease Philadelphia, PA: Saunders/Elsevier; 2010.

[15] Tipnis SR, Hooper NM, Hyde R, Karran E, Christie G, Turner AJ. A human homolog of angiotensin-converting enzyme. Cloning and functional expression as a captopril-insensitive carboxypeptidase. J Biol Chem. 2000;275(43):33238–43. Epub 2000/08/05. doi: 10.1074/jbc.M002615200 . 10924499 10.1074/jbc.M002615200

[16] Donoghue M, Hsieh F, Baronas E, Godbout K, Gosselin M, Stagliano N, et al A novel angiotensin-converting enzyme-related carboxypeptidase (ACE2) converts angiotensin I to angiotensin 1–9. Circ Res. 2000;87(5):E1–9. Epub 2000/09/02. . 10969042 10.1161/01.res.87.5.e1

[17] Al-Maghrebi M, Benter IF, Diz DI. Endogenous angiotensin-(1–7) reduces cardiac ischemia-induced dysfunction in diabetic hypertensive rats. Pharmacol Res. 2009;59(4):263–8. Epub 2009/01/27. doi: 10.1016/j.phrs.2008.12.008 ; PubMed Central PMCID: PMCPMC2682227. 19166939 10.1016/j.phrs.2008.12.008 PMC2682227

[18] Sampaio WO, Henrique de Castro C, Santos RA, Schiffrin EL, Touyz RM. Angiotensin-(1–7) counterregulates angiotensin II signaling in human endothelial cells. Hypertension. 2007;50(6):1093–8. Epub 2007/11/07. doi: 10.1161/HYPERTENSIONAHA.106.084848 . 17984366 10.1161/HYPERTENSIONAHA.106.084848

[19] Sampaio WO, Souza dos Santos RA, Faria-Silva R, da Mata Machado LT, Schiffrin EL, Touyz RM. Angiotensin-(1–7) through receptor Mas mediates endothelial nitric oxide synthase activation via Akt-dependent pathways. Hypertension. 2007;49(1):185–92. Epub 2006/11/23. doi: 10.1161/01.HYP.0000251865.35728.2f . 17116756 10.1161/01.HYP.0000251865.35728.2f

[20] Murphy PG, Myers DS, Davies MJ, Webster NR, Jones JG. The antioxidant potential of propofol (2,6-diisopropylphenol). Br J Anaesth. 1992;68(6):613–8. Epub 1992/06/01. . 1319189 10.1093/bja/68.6.613

[21] Mikawa K, Akamatsu H, Nishina K, Shiga M, Maekawa N, Obara H, et al Propofol inhibits human neutrophil functions. Anesth Analg. 1998;87(3):695–700. Epub 1998/09/05. . 9728856 10.1097/00000539-199809000-00039

[22] Chen J, Gu Y, Shao Z, Luo J, Tan Z. Propofol protects against hydrogen peroxide-induced oxidative stress and cell dysfunction in human umbilical vein endothelial cells. Mol Cell Biochem. 2010;339(1–2):43–54. Epub 2009/12/30. doi: 10.1007/s11010-009-0368-y . 20039104 10.1007/s11010-009-0368-y

[23] Luo T, Xia Z, Ansley DM, Ouyang J, Granville DJ, Li Y, et al Propofol dose-dependently reduces tumor necrosis factor-alpha-Induced human umbilical vein endothelial cell apoptosis: effects on Bcl-2 and Bax expression and nitric oxide generation. Anesth Analg. 2005;100(6):1653–9. Epub 2005/05/28. doi: 10.1213/01.ANE.0000150945.95254.D8 . 15920191 10.1213/01.ANE.0000150945.95254.D8

[24] Balyasnikova IV, Visintine DJ, Gunnerson HB, Paisansathan C, Baughman VL, Minshall RD, et al Propofol attenuates lung endothelial injury induced by ischemia-reperfusion and oxidative stress. Anesth Analg. 2005;100(4):929–36. Epub 2005/03/23. doi: 10.1213/01.ANE.0000147707.49192.88 . 15781500 10.1213/01.ANE.0000147707.49192.88

[25] Xia Z, Luo T, Liu HM, Wang F, Xia ZY, Irwin MG, et al L-arginine enhances nitrative stress and exacerbates tumor necrosis factor-alpha toxicity to human endothelial cells in culture: prevention by propofol. J Cardiovasc Pharmacol. 2010;55(4):358–67. Epub 2010/02/04. doi: 10.1097/FJC.0b013e3181d265a3 . 20125033 10.1097/FJC.0b013e3181d265a3

[26] Dworakowski R, Alom-Ruiz SP, Shah AM. NADPH oxidase-derived reactive oxygen species in the regulation of endothelial phenotype. Pharmacol Rep. 2008;60(1):21–8. Epub 2008/02/16. . 18276982

[27] Alvarez E, Rodino-Janeiro BK, Ucieda-Somoza R, Gonzalez-Juanatey JR. Pravastatin counteracts angiotensin II-induced upregulation and activation of NADPH oxidase at plasma membrane of human endothelial cells. J Cardiovasc Pharmacol. 2010;55(2):203–12. Epub 2009/12/17. doi: 10.1097/FJC.0b013e3181ce5f5a . 20010434 10.1097/FJC.0b013e3181ce5f5a

[28] Chen Z, Hu Z, Lu Z, Cai S, Gu X, Zhuang H, et al Differential microRNA profiling in a cellular hypoxia reoxygenation model upon posthypoxic propofol treatment reveals alterations in autophagy signaling network. Oxid Med Cell Longev. 2013;2013:378484 Epub 2014/01/24. doi: 10.1155/2013/378484 ; PubMed Central PMCID: PMCPMC3885199. 24454982 10.1155/2013/378484 PMC3885199

[29] Nicholson JK, Lindon JC, Holmes E. 'Metabonomics': understanding the metabolic responses of living systems to pathophysiological stimuli via multivariate statistical analysis of biological NMR spectroscopic data. Xenobiotica. 1999;29(11):1181–9. Epub 1999/12/22. doi: 10.1080/004982599238047 . 10598751 10.1080/004982599238047

[30] Xu JJ, Wang YL. Propofol attenuation of hydrogen peroxide-mediated oxidative stress and apoptosis in cultured cardiomyocytes involves haeme oxygenase-1. Eur J Anaesthesiol. 2008;25(5):395–402. Epub 2008/02/22. doi: 10.1017/S0265021508003542 . 18289444 10.1017/S0265021508003542

[31] Ferrario CM. New physiological concepts of the renin-angiotensin system from the investigation of precursors and products of angiotensin I metabolism. Hypertension. 2010;55(2):445–52. Epub 2009/12/23. doi: 10.1161/HYPERTENSIONAHA.109.145839 ; PubMed Central PMCID: PMCPMC2810712. 20026757 10.1161/HYPERTENSIONAHA.109.145839 PMC2810712

[32] Vickers C, Hales P, Kaushik V, Dick L, Gavin J, Tang J, et al Hydrolysis of biological peptides by human angiotensin-converting enzyme-related carboxypeptidase. J Biol Chem. 2002;277(17):14838–43. Epub 2002/01/30. doi: 10.1074/jbc.M200581200 . 11815627 10.1074/jbc.M200581200

[33] Der Sarkissian S, Huentelman MJ, Stewart J, Katovich MJ, Raizada MK. ACE2: A novel therapeutic target for cardiovascular diseases. Prog Biophys Mol Biol. 2006;91(1–2):163–98. Epub 2005/07/13. doi: 10.1016/j.pbiomolbio.2005.05.011 . 16009403 10.1016/j.pbiomolbio.2005.05.011

[34] Cao L, Xu L, Huang B, Wu L. Propofol increases angiotensin-converting enzyme 2 expression in human pulmonary artery endothelial cells. Pharmacology. 2012;90(5–6):342–7. Epub 2012/10/26. doi: 10.1159/000338754 . 23095677 10.1159/000338754

[35] Higuchi S, Ohtsu H, Suzuki H, Shirai H, Frank GD, Eguchi S. Angiotensin II signal transduction through the AT1 receptor: novel insights into mechanisms and pathophysiology. Clin Sci (Lond). 2007;112(8):417–28. Epub 2007/03/10. doi: 10.1042/cs20060342 . 17346243 10.1042/CS20060342

[36] Griendling KK, FitzGerald GA. Oxidative stress and cardiovascular injury: Part II: animal and human studies. Circulation. 2003;108(17):2034–40. Epub 2003/10/29. doi: 10.1161/01.CIR.0000093661.90582.c4 . 14581381 10.1161/01.CIR.0000093661.90582.c4

[37] Griendling KK, FitzGerald GA. Oxidative stress and cardiovascular injury: Part I: basic mechanisms and in vivo monitoring of ROS. Circulation. 2003;108(16):1912–6. Epub 2003/10/22. doi: 10.1161/01.CIR.0000093660.86242.BB . 14568884 10.1161/01.CIR.0000093660.86242.BB

[38] Gulcin I, Alici HA, Cesur M. Determination of in vitro antioxidant and radical scavenging activities of propofol. Chem Pharm Bull (Tokyo). 2005;53(3):281–5. Epub 2005/03/04. . 15744098 10.1248/cpb.53.281

[39] Kobayashi K, Yoshino F, Takahashi SS, Todoki K, Maehata Y, Komatsu T, et al Direct assessments of the antioxidant effects of propofol medium chain triglyceride/long chain triglyceride on the brain of stroke-prone spontaneously hypertensive rats using electron spin resonance spectroscopy. Anesthesiology. 2008;109(3):426–35. Epub 2008/08/23. doi: 10.1097/ALN.0b013e318182a903 . 18719440 10.1097/ALN.0b013e318182a903

[40] Bodelsson G, Sandstrom K, Wallerstedt SM, Hidestal J, Tornebrandt K, Bodelsson M. Effects of propofol on substance P-induced relaxation in isolated human omental arteries and veins. Eur J Anaesthesiol. 2000;17(12):720–8. Epub 2000/12/21. . 11122309 10.1046/j.1365-2346.2000.00749.x

