# Supplementary figures and images for: Propofol prevents human umbilical vein endothelial cell injury from Ang II-induced apoptosis by activating the ACE2-(1-7)-Mas axis and eNOS phosphorylation
Source: PLoS One. 2018 Jul 11;13(7):e0199373. doi: 10.1371/journal.pone.0199373 (PMC6040691; doi:10.1371/journal.pone.0199373)

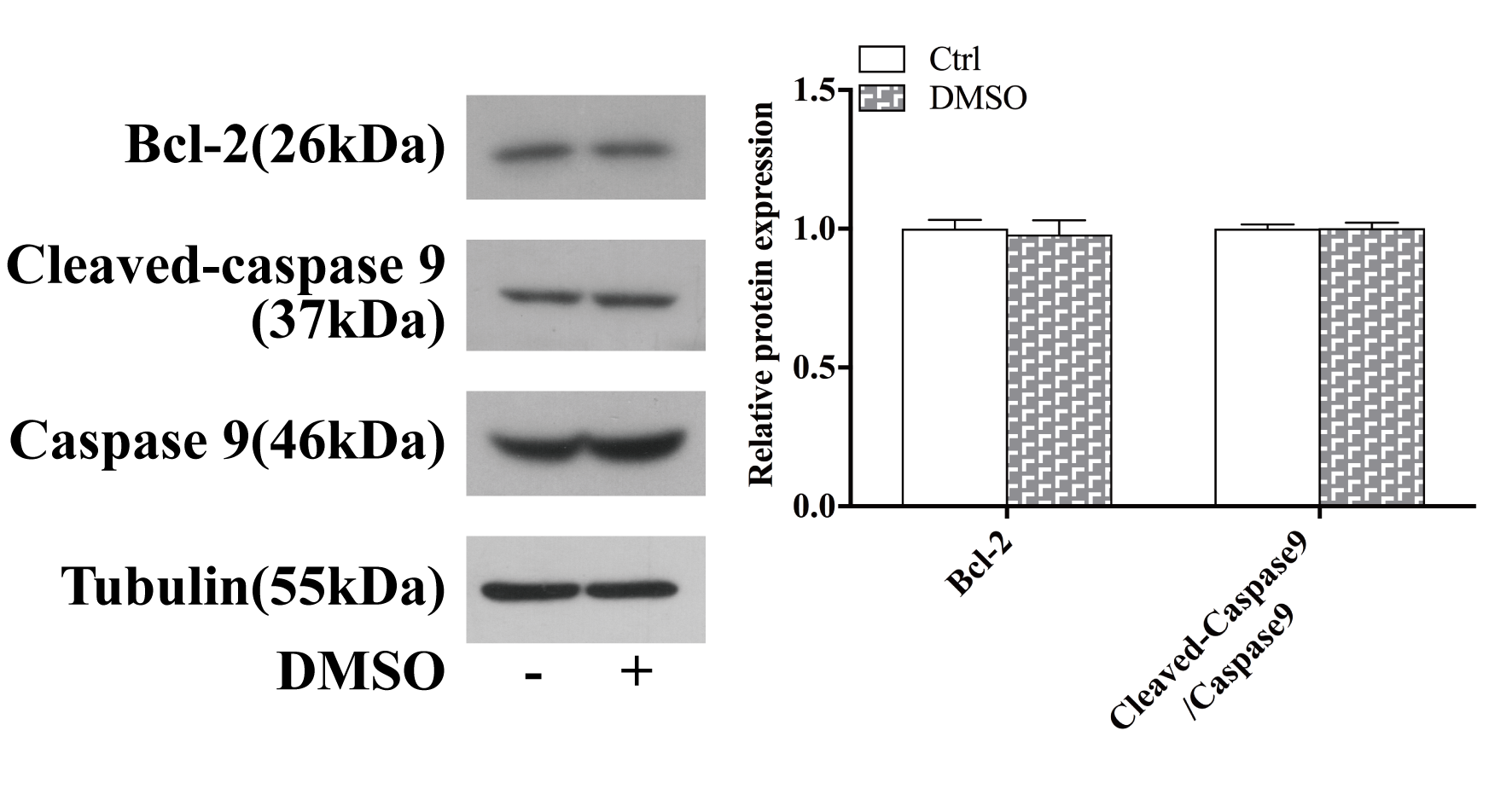

Supplement: S1 Fig — Cells were treated with or without 0.1% DMSO for 4h, and then cleaved-caspase 9, caspase 9, Bcl-2 were detected by Western blot analysis. The results are shown as the mean ± SD (n = 3). (TIF) [file pone.0199373.s001.tif]

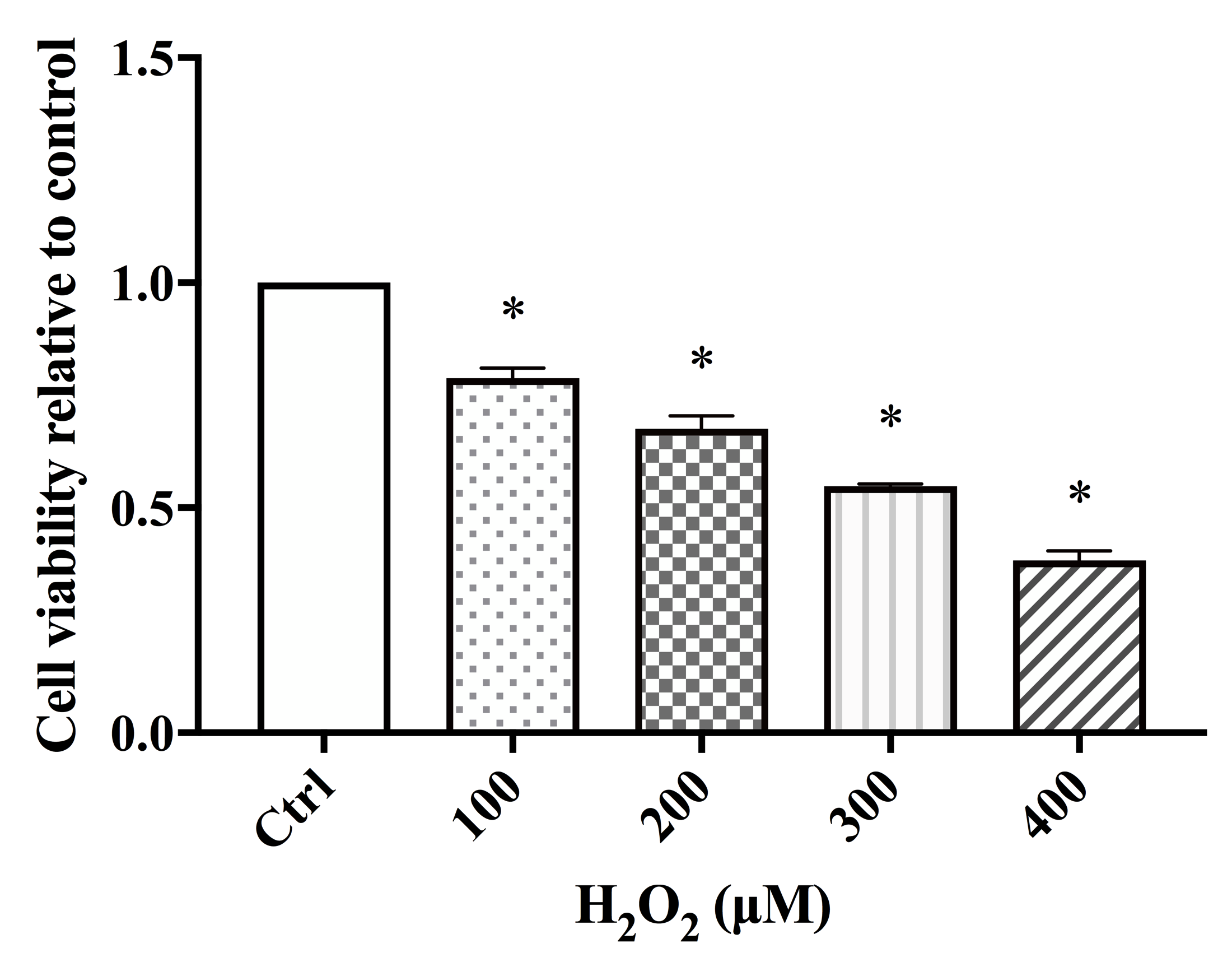

Supplement: S2 Fig — The cell viability was measured by CCK8 assay. HUVECs were incubated with different concentration of H2O2 for 4 h. The results are shown as means ± SD (n = 3). * P < 0.01, compared with control. (TIFF) [file pone.0199373.s002.tiff]

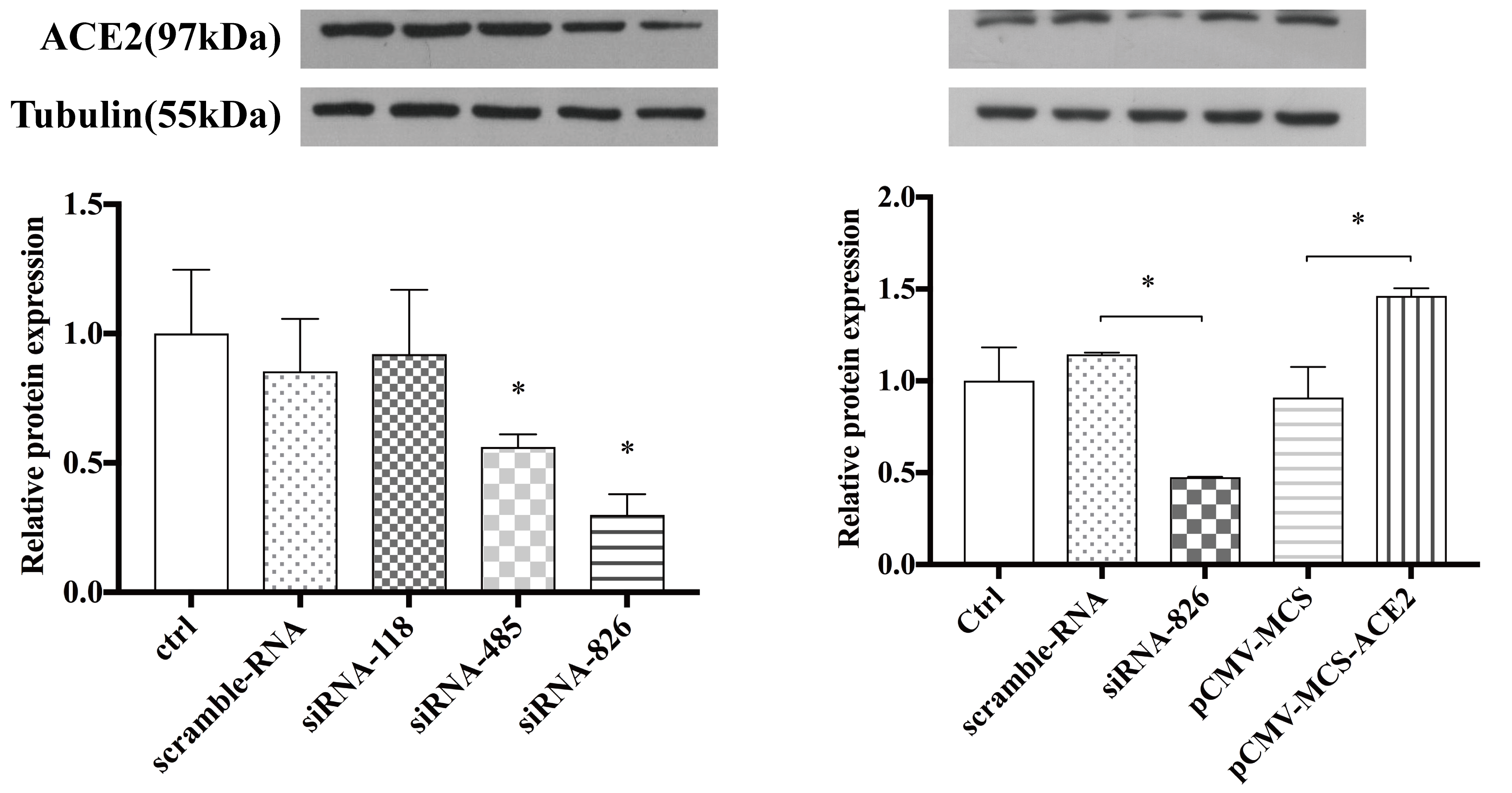

Supplement: S3 Fig — Western blot showed ACE2 protein expression which was knockout or overexpressed. The results are shown as means ± SD (n = 3). * P < 0.01, compared with control. (TIF) [file pone.0199373.s003.tif]

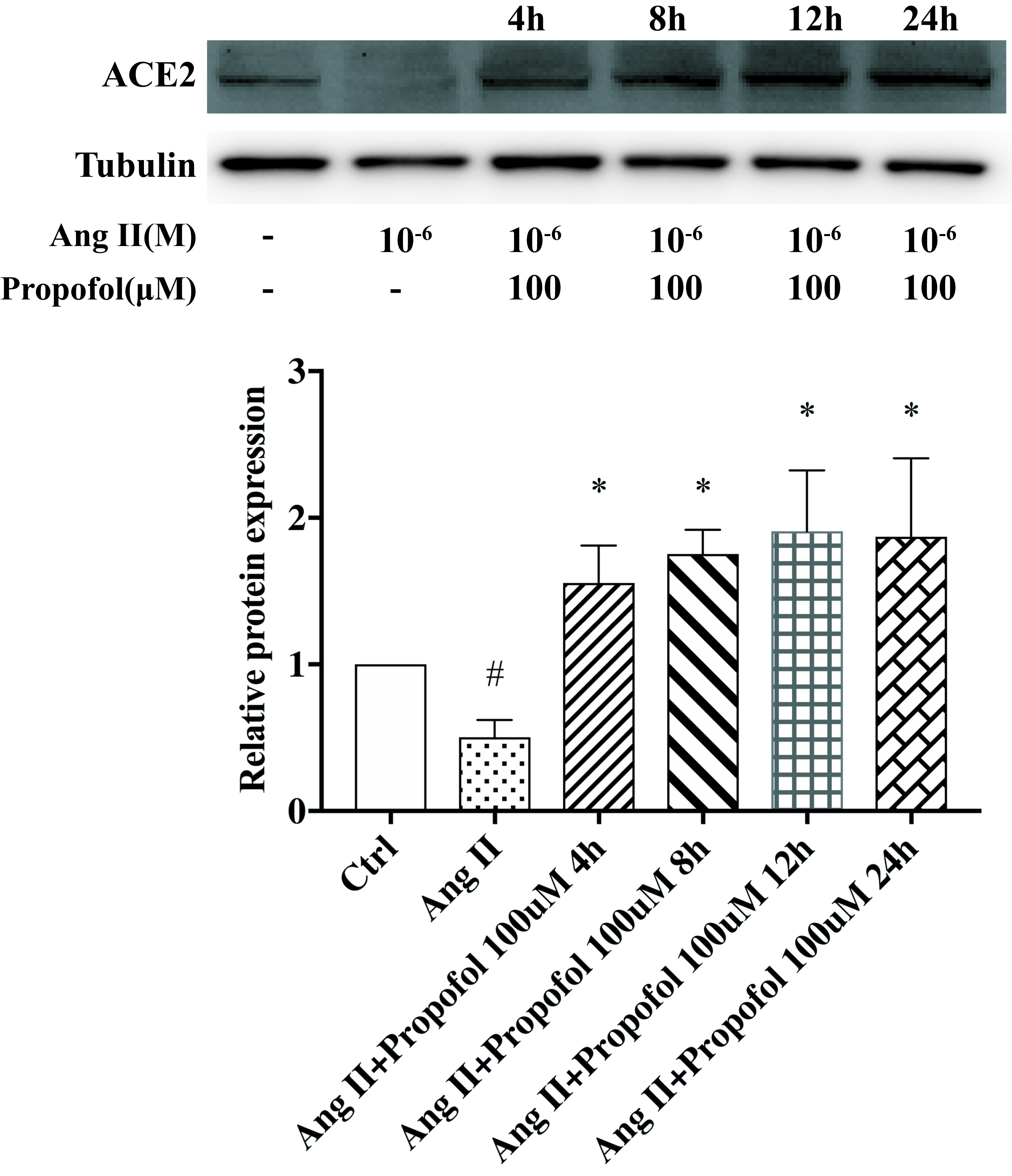

Supplement: S4 Fig — Western blot showed ACE2 protein expression which was treated with propofol in AngII injured HUVECs. The results are shown as means ± SD (n = 3). # P < 0.01, compared with control; * p<0.05 compared to the Ang II group. (TIF) [file pone.0199373.s004.tif]
